# Evaluating the relationship between emotional intelligence and cognitive disorders in patients with Multiple Sclerosis

**Published:** 2018-04-04

**Authors:** Mahsa Owji, Mohammad Ali Sahraian, Maryam Bidadian, Fereshteh Ghadiri, Farnaz Etesam, Amirreza Azimi, Abdorreza Naser Moghadasi

**Affiliations:** 1 Multiple Sclerosis Research Center, Neuroscience Institute, Tehran University of Medical Sciences, Tehran, Iran; 2 Department of Psychology, School of Humanities, Tarbiat Modares University, Tehran, Iran; 3 Department of Psychiatry, Baharloo Hospital, Tehran University of Medical Sciences, Tehran, Iran

**Keywords:** Emotional Intelligence, Cognitive Disorders, Multiple Sclerosis

## Abstract

**Background:** Emotional intelligence refers to a process through which an individual is not only capable of understanding his/her/others’ emotions, but also is able to manage them. Emotional intelligence can get disturbed due to different neurological diseases. Since cognition and emotion are directly and closely related, the present study aims to evaluate the relationship between emotional intelligence and cognitive disorders in patients with Multiple Sclerosis (MS).

**Methods:** The demographic data of 92 MS patients participating in this study were recorded. The emotional intelligence and cognitive disorders were studied using the Bradberry-Greaves, and MS Neuropsychological Questionnaire (MSNQ) tests, respectively.

**Results:** 16 men and 76 women were considered in this study. The mean age of the participants was 33.4 years, the mean duration of the disease was 6.8 years, the mean of Expanded Disability Status Scale (EDSS) was 1.97, the mean MSNQ was 21.58, and the mean emotional quotient (EQ) of the patients was 74.18. The MSNQ had a significant relationship with the total EQ and its sub-categories (P < 0.05).

**Conclusion:** This study showed that EQ and cognitive disorders are directly relevant to each other; as cognitive disorder increases, the EQ rate decreases. Therefore, cognitive rehabilitation might be effective in enhancing the EQ in these patients.

## Introduction

Emotional intelligence or emotional quotient (EQ) refers to a process through which an individual obtains the ability to perceive, control, and evaluate his/her/others’ emotions and manage them. This is an effective factor in adjusting with the surrounding environment.^1^ The EQ changes due to different neurological diseases{Hoffmann, 2010 #1}.^2^ Studies have been conducted on stroke and frontotemporal dementia (FTD), and it has been revealed that such diseases can be accompanied with behavioral and emotional disorders.^1^^,^^3^

The relationship between emotion and cognition is one of the important aspects of human communication with the surrounding world. According to Pessoa, the two issues are not separate from each other because the regions of the brain involved in processing emotion or cognition are the same.^4^

Multiple Sclerosis (MS) is a chronic autoimmune disease of the central nervous system (CNS) that involves the brain and spinal cord. MS is one of the most common and leading causes of physical disability in young adults. Many of the lesions in the brain are apparently asymptomatic on the routine physical examinations but they may cause subtle to prominent cognitive and behavioral changes in these patients. Although there are some old reports on the memory problems in MS, they were more or less ignored due to the greater attention of physicians to the physical aspect of the disease. In recent years, a large number of studies have demonstrated that the cognitive disorders are common among patients with MS and can involve different areas like memory, information processing, and executive functions.^5^^,^^6^

The relationship between emotional intelligence and cognitive disorders has not been adequately studied in these patients. Here we evaluated the relationship of EQ and cognitive problems in patients with MS in order to see if the cognitive deficits may or may not change the EQ in patients with MS.

## Materials and Methods

There were 92 eligible patients selected from the outpatient clinic. The inclusion criteria for the patients were relapsing–remitting MS (RRMS), minimum education level of high school diploma, and the age of the patient being between 18 to 50 years old. The exclusion criteria were any attacks or treatments with corticosteroids in the previous one month, and any other accompanying diseases.

The Bradberry-Greaves’ test was used for examining the EQ in the patients. This test was first introduced by Bradberry and Greaves in 2005.^7^ It has 28 parts further divided into 5 separate parts. These independent parts include general EQ, self-awareness, self-management, social awareness, and relationship management. The test has previously been translated into Persian and validated.^8^^,^^9^

The MS Neuropsychological Questionnaire (MSNQ) test was used for studying the cognitive disorders. It was revealed that MSNQ is directly associated with cognitive disorders in patients with MS.^10^^,^^11^

The SPSS software (version 16, SPSS Inc., Chicago, IL, USA) was used for the statistical analysis of the data. Since the variables had not been normally distributed, the Spearman correlation test was applied for studying the relationship between the variables.

The ethics committee of the Tehran University of Medical Sciences approved the study on the basis of the Helsinki declaration.

## Results

There were 16 men and 76 women included in this study. The mean age of the participants was 33.4 years (Men: 34.4 years, and women: 33.2 years). Their age was in the range of 21-50 years old.

The mean duration of the disease was 6.8 years (Men: 6 years, and women: 6.9 years). The range of the duration of the disease was between 1 to 20 years. The mean Expanded Disability Status Scale (EDSS) of the patients was 1.97 years (men: 2.46 years, and women: 1.86 years). The range of EDSS was from 0 to 4 (Table 1).

**Table 1:** Demographic characteristics of the patients

**Characteristics**	**Total**	**Men**	**Women**
Number of patients	92	16	76
Mean age of patients (year)	33.40	34.40	33.20
Mean duration of disease (year)	6.80	6.00	6.90
Mean of EDSS	1.97	2.46	1.89

EDSS: Expanded Disability Status Scale

The mean MSNQ of the patients was 21.58 (Men: 18.50, and women: 22.22).

The mean EQ of the patients was 74.18 (Men: 76.69, and women: 73.66). 

The mean self-awareness of the patients was 80.79 (Men: 82.56, and women: 80.42) without any significant statistical differences (P = 0.400).

The mean self-management of the patients was 64.27 (Men: 66.63, and women: 63.78) without any significant statistical differences (P = 0.560).

The mean relationship management of the patients was 73.97 (Men: 77.25, and women: 73.28) without any significant statistical differences (P = 0.270).

The mean social management of the patients was 79.09 (Men: 79.44, and women: 79.01) without any significant statistical differences (P = 0.880) (Table 2).

**Table 2:** The detailed characteristics of Multiple Sclerosis neuropsychological questionnaire (MSNQ), emotional quotient (EQ) and each of its subcategories

**Variable**	**Total score**	**Men**	**Women**
MSNQ (mean)	21.58	18.50	22.22
EQ (mean)	74.18	76.69	73.66
Self-awareness (mean)	80.79	82.56	80.42
Self-management (mean)	64.27	66.63	63.78
Relationship management (mean)	73.97	77.25	73.28
Social management (mean)	79.09	79.44	79.01

MSNQ: Multiple Sclerosis neuropsychological questionnaire; EQ: Emotional quotient

**Figure 1:**
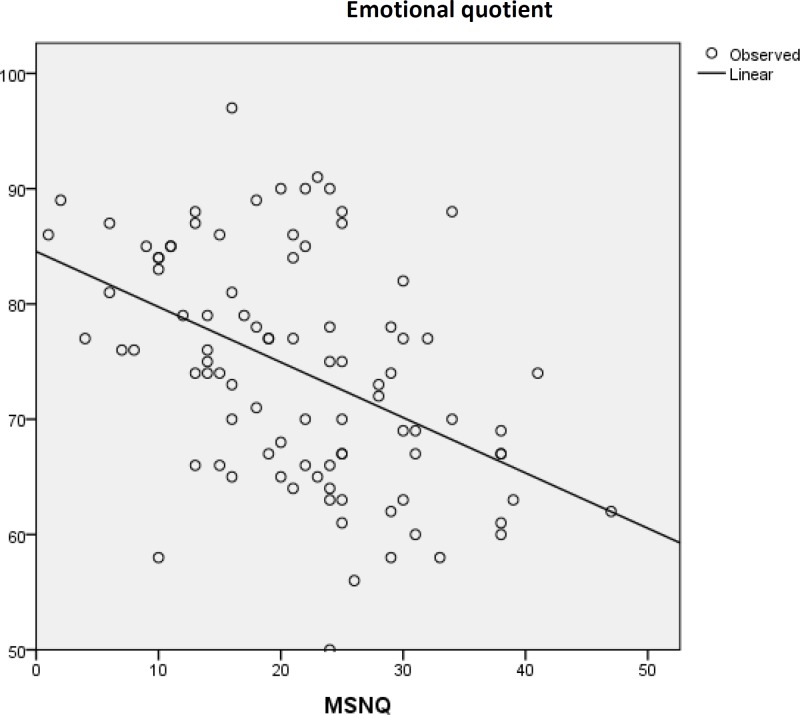
The significant relationship between Multiple Sclerosis neuropsychological questionnaire (MSNQ) and Emotional quotient (EQ)

The MSNQ and total EQ for each of the sub-categories including self-awareness, self-management, relationship management, and social management were significantly related (P-values were < 0.001, 0.013, < 0.001, and 0.006, respectively) (Figure 1).

## Discussion

Our study showed that MSNQ had a direct relationship with EQ and each of the five scales. This means that a patient with more cognitive disorders proportionally suffers from EQ disorder. This is the first study on the relationship between EQ and cognitive impairment in the patients with MS.

The previous studies showed that EQ has a direct relationship with different health components, such as stress and well-being.^12^^-^^14^ Since the EQ disorder in the MS patient is significantly more prevalent than the normal patient,^15^ this study could improve our knowledge about the various aspects of EQ disorder in the patients with MS.

Our findings can be interpreted from different approaches. From the view of cognitive psychology, the cognitive abilities are necessary for managing emotional experiences.^16^

It has also been demonstrated that the cognitive behavioral treatment methods are effective in treating emotional disorders.^17^ Excluding the psychological aspects, it seems that the main reason for the close relationship between emotion and cognition is because of the shared neural networks between them.^4^^,^^18^

As previously mentioned, cognitive disorders are quite common among patients with MS and seem to occur in the initial phases of the disease.^19^^,^^20^ On the other hand, mood and emotional disorders are commonly known in patients with MS.^21^

Few studies have been carried out on the relationship between emotional and cognitive disorders in MS patients; however, these studies showed the association between depression and cognitive disorders in these patients.^22^^-^^24^ These evidence can explain our findings, which can play an important role in the treatment of the patients. As the study shows, cognitive disorder is an important factor for the quality of life (QOL) in the patients.^25^^,^^26^ Therefore, identifying the relationship between EQ and cognition in the patients with MS can help us devise the treatment methods. Perhaps, some problems of the MS patients are due to the EQ disorder. Since EQ and cognition are closely related, providing cognitive rehabilitation might be effective in the MS patients’ recovery.

## Conclusion

The present study shows that the cognitive and EQ disorders in patients with MS are closely and directly related. Thus, one of the factors in enhancing the emotional status of the patients can be the treatment of the cognitive disorders.

## Notes:

**How to cite this article:** Owji M, Sahraian MA, Bidadian M, Ghadiri F, Etesam F, Azimi A, et al. Evaluating the relationship between emotional intelligence and cognitive disorders in patients with Multiple Sclerosis. Iran J Neurol 2018; 17(2): 78-81.
